# Association of Healthcare and Aesthetic Procedures with Infections Caused by Nontuberculous Mycobacteria, France, 2012‒2020 (response)

**DOI:** 10.3201/eid2806.220686

**Published:** 2022-06

**Authors:** Côme Daniau, Anne Berger-Carbonne, Emmanuelle Cambau

**Affiliations:** Santé Publique France, Saint-Maurice, France (C. Daniau, A. Berger-Carbonne);; Assistance Publique Hôpitaux de Paris, Centre National de Référence des Mycobactéries et de la Résistance des Mycobactéries aux Antituberculeux, Paris, France (E. Cambau)

**Keywords:** nontuberculous mycobacteria, bacteria, nontuberculous mycobacteria infections, tuberculosis and other mycobacteria, respiratory infections, healthcare-associated infections, aesthetic procedure‒associated infections, comparative genomics, epidemiology, case reports, retrospective study, France

**In Response:** We thank McNamara and colleagues for their commentary (*1*) on our article (*2*). We agree that early recognition of potential outbreaks of healthcare-acquired infections (HAIs) caused by nontuberculous mycobacteria (NTM is crucial for controlling the spread of those diseases that pose human and financial burdens. Because NTM are not transmissible from human to human and are not on the list of highly pathogenic bacteria, reporting of NTM infections is not mandatory. Consequently, specific reporting methods are needed to organize this information. In France, we had the opportunity to combine the national early warning response system (EWRS) for HAIs diagnosed in healthcare facilities, computerized since 2011, and the networking of clinical microbiologists with the National Reference Centre for Mycobacteria and Resistance of Mycobacteria to Anti-Tuberculosis Agents (CNRMyRMA). In addition, the French Public Health Agency, which receives data and coordinates the response in the EWRS, directly communicates with the CNRMyRMA, and they can make decisions in common with other professionals and health authorities involved. For each notified case of NTM HAI (from EWRS or from an isolate sent to CNRMyRMA), active research of other previous cases is recommended. However, it is often not easy, because of diagnosis and notification delays, to investigate associated practices and potential environmental sources. After 2 cases are reported in EWRS, investigations can be focused on common expositions or procedures, leading to targeted environmental specimens.

We believe that underestimations of the number of cases in France can be improved by increasing awareness of healthcare professionals through publications describing the risk factors associated with NTM HAI and by triggering clinical and microbiological vigilance through networks and registers. We hope that our article (*2*) together with this comment will help emphasize the value of the public health approach to NTM infections.
